# Suitability of Immobilized Systems for Microbiological Degradation of Endocrine Disrupting Compounds

**DOI:** 10.3390/molecules25194473

**Published:** 2020-09-29

**Authors:** Danuta Wojcieszyńska, Ariel Marchlewicz, Urszula Guzik

**Affiliations:** Institute of Biology, Biotechnology and Environmental Protection, Faculty of Natural Science, University of Silesia in Katowice, Jagiellońska 28, 40-032 Katowice, Poland; danuta.wojcieszynska@us.edu.pl (D.W.); ariel.marchlewicz@us.edu.pl (A.M.)

**Keywords:** EDCs, hormones, degradation, immobilization, microorganisms

## Abstract

The rising pollution of the environment with endocrine disrupting compounds has increased interest in searching for new, effective bioremediation methods. Particular attention is paid to the search for microorganisms with high degradation potential and the possibility of their use in the degradation of endocrine disrupting compounds. Increasingly, immobilized microorganisms or enzymes are used in biodegradation systems. This review presents the main sources of endocrine disrupting compounds and identifies the risks associated with their presence in the environment. The main pathways of degradation of these compounds by microorganisms are also presented. The last part is devoted to an overview of the immobilization methods used for the purposes of enabling the use of biocatalysts in environmental bioremediation.

## 1. Introduction

The development of modern tools for the separation and identification of chemical substances has drawn attention to the so-called micropollution of the environment. Many of these contaminants belong to the emergent pollutants class. According to the Stockholm Convention they are characterized by high persistence, are transported over long distances in the environment through water, accumulate in the tissue of living organisms and can adversely affect them [1]. Substances meeting these criteria include pharmaceuticals, the presence of which are not only found in hospital or municipal sewage but also in Arctic water or drinking water [1,2]. In many laboratories, new methods for the degradation of these compounds are being developed, with the use of microorganisms that have an increased potential for the degradation of these compounds. Biological treatment is a promising technology due to its low cost and reduced energy requirements [3,4]. However, the main problem is the survival of these microorganisms in the complex microbial consortia of wastewater treatment plants [5]. Hence, more and more attention has been paid to immobilized systems, in which these strains are protected by binding them to a carrier [4,6,7]. The use of microorganism immobilization in the drug’s biodegradation not only protects them from harmful environmental conditions, but also leads to an increase in the intensity of the biotransformation processes as a result of the local thickening of the biomass and a higher tolerance of the bacterial cells to these pharmaceuticals [4,7]. The intensive development of immobilization methods has led to the discovery of many carriers and immobilization techniques [7,8]. The new techniques differ in the level of difficulty of the procedure used, cost-effectiveness, efficiency of the processes carried out in the immobilized systems and the usefulness in the immobilization of the microorganisms. For this reason, the purpose of this work is to review the achievements to date in the field of using immobilized systems in the endocrine disrupting compounds biodegradation processes. This will systematize the existing knowledge about the subject and indicate the advantages and disadvantages of the methods used, as well as the needs and perspectives for the further development of immobilization methods.

## 2. Endocrine Disrupting Compounds (EDCs) in the Environment and Associated Risks

Endocrine disrupting compounds (EDCs) are natural or synthetic substances that, when introduced into the environment, affect the endocrine and homeostatic system of non-target organisms by disabling their communication and response to environmental stimuli. These include estrogens- natural and synthetic female and male hormones that play an important role in both the reproductive and the non-reproductive systems [3,6,9]. Estrone, estriol and 17β-estradiol are natural estrogens. The latter is considered to be the strongest among the endocrine factors in the environment [10]. Synthetic estrogens have been divided into steroidal, such as 17α-ethynylestradiol and 3-methyl ether of ethynylestradiol, used as ingredients of birth control pills, and nonsteroidal estrogens such as diethylstilbestrol used to prevent miscarriages [3,7,11]. Human use of the latter was banned in 1972 due to its negative effects, however, it is still used in China as a growth promotor for terrestrial livestock or fish [11]. The presence of estrogens has been reported in many aquatic environments (Table 1).

One of the main sources of estrogens in a highly urbanized environment is urine and faeces getting into wastewater. It has been shown that annually 33 and 49 tonnes of estrogen excreted from farms in the European Union and the United States, respectively, are released into the environment [12,20]. In human and animal organisms, estrogens are usually hydroxylated or conjugated with glucuronide or sulfate hence these forms most often get into wastewater. It is estimated that about 90% of urine estrogens are in the form of conjugates. Most estrogen conjugates are more polar than parent molecules, so they can be more mobile in the environment. Despite the fact that conjugates have low estrogenic potential, they can be chemically or enzymatically hydrolysed to active forms in the environment. For example, the *Escherichia coli* strain KCTC 2571 has β-glucuronidase, which cleaves glucuronide moieties, releasing free estrogen [20,21].

As a result of the operation of sewage treatment plants, 19–94% of estrogens are biodegraded and biotransformed. Typically, concentrations in the range of 1–3 ng/L are observed in the effluents from treatment plants [3]. Although these compounds occur in the environment in concentrations of ng/L, it is known that they can have a strong negative impact in amounts below 1 ng/L because they have a high affinity for nuclear estrogen receptors. This biological activity can lead to low levels of estrogen in the environment causing endocrine disorders in the wildlife population. Negative effects on development, sex differentiation, reproduction of different animal species and production of vitellogenin have been documented [3,4,7,11,12]. Intersexual disorders in fish in Europe, and a negative impact on the sexual behaviour of frogs in suburban areas in the United States have been observed [12]. In addition, it has been shown that 17β-estradiol, at concentrations of 0.1–1 ng/L, causes reproductive disorders in aquatic animals. Disrupting in the development, spawning and fertility of Japanese medaka (*Oryzias latipes*) was showed at low estrogen concentrations [20]. An increase in osteoblast activity was also detected in fish such as goldfish and wrasse, which resulted in the mobilization and mineralization of fish scales [22]. The presence of this compound in the environment may cause disturbances in the secretion of endogenous gonadal sex hormone in humans and reduce sperm production, as well as increase the incidence of cancer [6]. In addition, it has been observed that often the response to estrogens is dependent on the age of the organisms. For instance, adults of common roach showed a slight response to the presence of estrogens, while exposure to hormonal compounds during the embryonic period caused 100% feminization in fish. In toxicological studies at various developmental stages, Atlantic salmon showed a higher level of vitellogenin mRNA in the presence of EDCs in all stages, except in embryos. In addition, embryos were only sensitive to the highest levels of 17α-ethynylestradiol while the remaining stages responded to the intermediate and highest doses not only of 17α-ethynylestradiol, but also 17β-estradiol, and nonylphenol. Moreover, fry have been shown to be the most sensitive of the salmon life stage on EDCs [23]. Czarny et al. [24] showed a dose-dependent effect of estrogens on the growth of cyanobacteria *Microcystis aeruginosa*. Concentrations below 1 mg/L did not affect the growth of cyanobacteria, whereas concentrations of hormones above 10 mg/L inhibited their growth. This is due to the phenomenon of hormesis. Low hormone concentrations activate the repair functions of cyanobacteria and induce the activity of proteins involved in them. Higher concentrations damage the maintenance and repair functions of cells. It was also observed that the effect of chronic toxicity depended on the hormone used and it decreased according to the series: 17-α-ethynylestradiol > progesterone > 17ß-estradiol > 5-pregnen-3β-ol-20-one > testosterone > estrone> levonorgestrel > estriol. Moreover, the hormone mixture caused a significant increase in toxicity compared to the same concentrations of individual hormones. The range of EC_50_ values obtained for the mixture of hormones and individual hormones were 56.66–166.83 mg/L and 88.92–355.15 mg/L, respectively (according to the EU classification, pollutants with EC_50_ values of 10–100 mg/L are harmful to aquatic organisms). The negative impact of hormones on cyanobacteria is connected with the rapid penetration of hormones into cells and the impact on photosynthetic activity by interfering with the flow of electrons through the photosystem II [24]. A significant increase in the toxicity of the mixture compared to individual substances may translate into more serious ecological effects. This observation is all the more important because of the environment where we usually deal with mixtures of hormones. Moreover, estrogens can accumulate in the food chain and the effects may appear in the next generation, threatening the health of the human population [4,11,24]. Also, the World Health Organization classified estrogens as group 1 carcinogens and some of their catechol metabolites such as 4-hydroxyestrone are also carcinogens [12,25].

Another group of endocrine substances that can be found in the environment are androgens. Like estrogens they can interfere with the normal functioning of the endocrine system of non-target organisms in trace concentration and they have many sources from which they get into the water, and due to filtration they also get into underground water. One of the most commonly identified androgens in the environment is testosterone, its presence in the environment may cause a high percentage of males in the aquatic environment, appearance of male secondary sex characteristics in female fish, inhibition of vitellogenin induction, reduction of reproductive capacity and masculinization in mammals [26]. For example, defeminisation has been observed in fathead minnows that have been exposed to wastewater from cattle farms [27]. Moreover, it has been showed that male Atlantic salmon exhibit an odorant response to testosterone at extremely low concentration (0.003 ng/L) [28].

There are also substances in the environment that disturb the action of endogenous compounds due to the similarity of their structure to hormones (chemicals mimic hormones), such as bisphenol A, 4-nonylphenol, parabens and phthalate, as well as antibacterial triclosan [29,30]. Moreover, some mycotoxins, such as zearalenone produced by several *Fusarium* spp., have strong estrogenic activity [31]. These compounds are resistant to microbiological degradation and their duration in sediments can be up to 40 years and, similar to hormonal substances, sometimes they can be food contaminants and thus, they can cause reproductive and developmental disorders of aquatic animals and humans [29,31]. Kriszt et al. [31] showed that zearalenone significantly increased the uterus weight in a dose dependent manner and upregulated complement component 2, calbindin-3 expression, and decreased apelin and aquaporin 5 mRNA levels. This is due to the high affinity of zearalenone for estrogen receptors. Moreover, this compound and its metabolite α-zearalenol stimulate the proliferation of MCF-7 human adenocarcinoma cells [31]. Nonylphenol may cause various disorders of the male reproductive system, such as a reduction of testicle size and decrease of sperm production in *Oncorhynchus mykiss*. This mimic hormone was also observed to induce the proliferation of the human breast cancer cell line [32]. Due to the prevalence of endocrine compounds and other pharmaceuticals in the environment and their possible negative effect on non-target organisms, more and more attention is being paid to the methods of their removal from the environment.

## 3. Microbiological Degradation of EDCs

**Figure 1:**
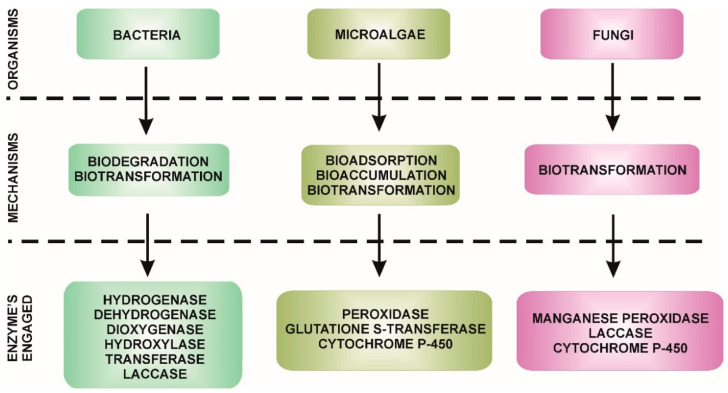
The main mechanisms for removing EDCs [3,9,26,30].

In developed countries, where the problem of endocrine substances is the greatest, sewage treatment plants play a key role in their removal. Organisms involved in the removal of estrogens from the environment include bacteria, as well as fungi and microalgae (Figure 1) [11].

Bacteria that degrade these hormones belong to genera such as *Bacillus*, *Virgibacillus*, *Novosphingobium*, *Rhodococcus*, *Acinetobacter*, *Pseudomonas*, *Sphingomonas*, *Enterobacter*, *Klebsiella*, *Aeromonas*, *Comamonas*, *Thauera*, *Deinococcus*, *Stenotrophomonas* and cyanobacteria [3,4,6,7,10,12,25,28,31,33,34,35,36,37,38,39]. *Raphidocelis subcapitata*, *Chlorella vulgaris*, *Chlorococcus* and *Scenedesmus quadricauda* belong to algae capable of removing hormones [11,26,40,41]. Fungi from genus *Trichoderma*, *Trametes*, *Phanerochaete*, *Pleurotus*, *Aspergillus*, *Lentinula*, *Drechslera*, *Curvularia*, *Penicillium*, *Acremonium*, *Fusarium*, *Gibberella*, *Beauvaria*, *Doaratomyces*, *Scopulariopsis*, *Chaetomidium*, *Thielavia*, *Neurospora*, *Absidia*, *Cunninghamella*, *Actinomucor*, *Rhizopus*, *Phycomyces*, *Mortierella*, *Circinella* and *Ganoderma* (Table 2) were also described as being capable of hormone biodegradation/biotransformation [22,30,42,43,44,45,46].

Bacteria that degrade these hormones belong to genera such as *Bacillus*, *Virgibacillus*, *Novosphingobium*, *Rhodococcus*, *Acinetobacter*, *Pseudomonas*, *Sphingomonas*, *Enterobacter*, *Klebsiella*, *Aeromonas*, *Comamonas*, *Thauera*, *Deinococcus*, *Stenotrophomonas* and cyanobacteria [3,4,6,7,10,12,25,28,31,33,34,35,36,37,38,39]. *Raphidocelis subcapitata*, *Chlorella vulgaris*, *Chlorococcus* and *Scenedesmus quadricauda* belong to algae capable of removing hormones [11,26,40,41]. Fungi from genus *Trichoderma*, *Trametes*, *Phanerochaete*, *Pleurotus*, *Aspergillus*, *Lentinula*, *Drechslera*, *Curvularia*, *Penicillium*, *Acremonium*, *Fusarium*, *Gibberella*, *Beauvaria*, *Doaratomyces*, *Scopulariopsis*, *Chaetomidium*, *Thielavia*, *Neurospora*, *Absidia*, *Cunninghamella*, *Actinomucor*, *Rhizopus*, *Phycomyces*, *Mortierella*, *Circinella* and *Ganoderma* (Table 2) were also described as being capable of hormone biodegradation/biotransformation [22,30,42,43,44,45,46].

The biodegradation processes of estrogens with the participation of bacteria occurs under both aerobic and anaerobic conditions, in both monosubstrate conditions and in cometabolic cultures [3,7,21,33,34,35]. Fernandez et al. [3] observed the biotransformation of 17β-estradiol to estrone under both aerobic and anaerobic conditions by bacteria isolated from deep sea sediments of mud volcanoes of the Gulf of Cadiz. The appearance of three intermediates of conjugated estrogen (17α-estradiol-3-sulfate) degradation has also been observed in both aerobic and anaerobic environments. However, degradation occurs much faster in an aerobic environment. Moreover, the main degradation mechanism varied depending on the oxygen limitation. Under aerobic conditions, the reaction initiating degradation was the oxygenation of position 17 of the 17α-estradiol-3-sulfate ring, which led to estrone-3-sulfate, while under anaerobic conditions the first degradation step was the deconjugation of the thioester bond at position 3, resulting in 17α-estradiol. In the second stage of degradation, the appearance of estrone was observed in both aerobic and anaerobic conditions [21].

As well as the degree of oxygenation, microbial degradation of hormones depends on the type and concentration of the endogenous substance, temperature, amount of suspended particles, age of activated sludge and additional carbon sources. A reduction of estradiol degradation was observed in the presence of glucose, sodium acetate and sodium citrate, whereas in the presence of methanol degradation was faster. Moreover, it was shown that the degradation rate depended on the carbon to nitrogen ratio - the lower it was, the faster the estrogen decomposition was. In addition, 17β-estradiol is usually degraded faster while estrone and synthetic estrogens are decomposed slower [34,47]. Moreover, Wang et al. [37] noted the synergistic effect of mixture strains during 17β-estradiol degradation. While individual strains degraded to 86% of the hormone, the mixture of strains almost completely degraded 17β-estradiol. Synergy is caused by the cross-metabolism of strains, which allows better use of the intermediates. This is indicated by the analysis of intermediates in a mixed strain culture, which showed the appearance of two organic acids. These compounds were not identified during degradation of individual strains [37].

Due to the complicated, multi-ring structure of estrogens, they are degraded under aerobic conditions in different pathways, depending on which ring is cleaved first and in which position. Usually the first step of 17β-estradiol degradation is its dehydrogenation to estrone. This reaction is of great environmental significance as this reduces the total estrogenic activity due to the oxygenation of the phenol group of 17β-estradiol to ketone moiety [6,10,22,35,38]. Xiong et al. [38], during genome analysis of *Deinococcus actinosclerus* SJTR1, found 18 genes encoding short-chain dehydrogenase/reductase (SDR), probably engaged in transformation 17β-estradiol to estrone. Estrone can be degraded in various pathways, during estrone metabolism by *Rhodococcus hoagii* BH2–1. Pratush et al. [48] identified two intermediates: 3-hydroxyandrosta-5,7,9(11)-trein-17-one and androsta-1,4,6-triene-3,17-dione, which were further metabolized. The mechanism of estrone degradation by cyanobacterial species is probably totally different. Sami and Fatma [49] postulate that in this process laccase is engaged. One of the known degradation pathways of estrone is the 4,5-seco pathway with a characteristic metabolite—pyridinestrone acid—which has no estrogenic activity. This transformation pathway was observed in estrone-degrading alphaproteobacterial, e.g., in the *Novosphingobium* sp. SLCC strain, which was isolated from the activated sludge of Dihua Sewage Treatment Plant in Taipei, and in the *Sphingomonas* sp. strain KC8. In the first step of degradation, estrone is hydroxylated with estrone 4-hydroxylase and the resulting 4-hydroxyestrone is cleaved by 4-hydroxyestrone 4,5-dioxygenase. Analysis of the metabolites also showed that estratetraenol formed during degradation with the SLCC strain, which is a characteristic degradation product of 17β-estradiol in biological sewage treatment plants [12,25]. 15 extradiol dioxygenase genes have been identified in the genome of the KC8 strain. The *oecC* cluster, which is responsible for the induction of active 4,5-dioxygenase, probably plays an important role in the catabolism of estrogens [25]. Wu et al. [50] noted that a *meta* cleavage product is converted by 2-oxoacid oxidoreductase to 4-norestrogen-(10)-en-3-oyl-CoA. Further transformations include sequences of ester intermediate reactions leading to hydrolytic cleavage of the B ring. Hydrolysis by 2-hydroxycyclohexanecarboxyl-CoA dehydrogenase is facilitated by the deficiency of electrons on the carbonyl carbon of the B ring. The end product of the pathway is 9,17-dioxo-1,2,3,4,10,19-hexanorandrostan-5-oic acid (HIP) [50]. Similarly, 17β-estradiol degradation is carried out by the *Acinetobacter strain* DSSKY-A-001. However, after cleavage of the ring to 4-(3α-methyl-3,7-dodecane-6H-cyclopentadiene[α]naphthalene-6-Subunit)-2-methoxy-3-butenoic acid, no re-closure of the ring to the heterocyclic end product- pyridinestrone acid was observed, as demonstrated in the typical 4,5-seco pathway. The obtained product is further oxidized to (*Z*)-8-(7α-methyl-1n-5-dioxo-octahydro-1*H*-inden-4-yl)-2o-6-dioxy-4-butenoic acid with the participation of the B ring cleaving oxygenase. In addition, hydroxylation at position 4 of the 17β-estradiol ring and conversion of 4-hydroxy-17β-estradiol to 4-hydroxyestrone were also observed [10,50].

Wang et al. [37] described 17β-estradiol degradation by a mixed culture of *Rhodococcus equi* DSSKP-R-001 and *Comamonas testosteroni* QYY20150409 and by individual strain. These authors showed that each strain degrades the hormone in a different pathway. 17β-estradiol degradation with the use of the strain QYY20150409 led through 2,6-dihydroxy-8-(7α-methyl-1,5-dioxooctahydro-inden-4-yl)-7-oxoocata-2,4- dienoic acid to succinic acid and 3-(7α-methyl-1,5-dioxooctahydro-1H-inden-4-yl)propanal. The strain DSSKP-R-001 degraded 17β-estradiol to 5-hydroxyhex-2-enedioic acid. 

**Figure 2:**
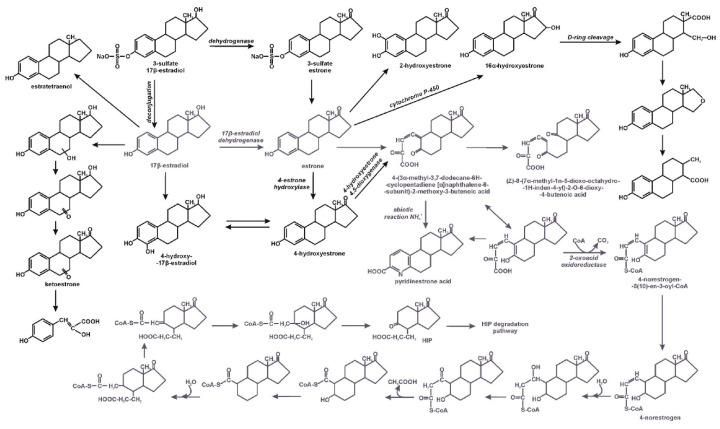
Estrogens biodegradation by bacteria; the 4,5-seco pathway reactions are shown in blue; HIP–9,17-dioxo-1,2,3,4,10,19-hexanorandrostan-5-oic acid [10,12,21,25,50].

**Figure 3:**
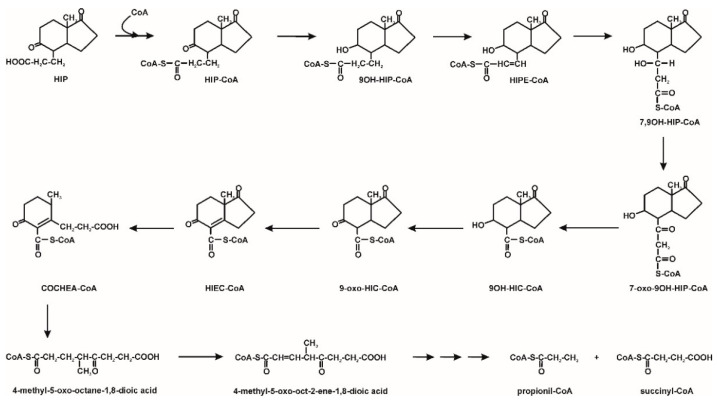
HIP degradation pathway; HIP—9,17-dioxo-1,2,3,4,10,19-hexanorandrostan-5-oic acid; HIP-CoA–9,17-dioxo-1,2,3,4,10,19-hexanorandrostan-5-oic acid CoA ester; 9OH-HIP-CoA–9-hydroxy-17-oxo-1,2,3,4,10,19-hexanorandrostan-5-oic acid CoA ester; HIPE-CoA–9-hydroxy-17-oxo-1,2,3,4,10,19-hexanorandrost-6-en-5-oic acid CoA ester; 7,9OH-HIP-CoA–7,9-dihydroxy-17-oxo-1,2,3,4,10,19-hexanorandrost-6-en-5-oic acid CoA ester; 7-oxo-9OH-HIP-CoA–7-oxo-9-hydroxy-17-oxo-1,2,3,4,10,19-hexanorandrost-6-en-5-oic acid CoA ester; 9OH-HIC-CoA–9α-hydroxy-17-oxo-1,2,3,4,5,6,10,19-octanorandrostan-7-oic acid CoA ester; 9-oxo-HIC-CoA–9-oxo-17-oxo-1,2,3,4,5,6,10,19-octanorandrostan-7-oic acid CoA ester; HIEC-CoA–9,17-dioxo-1,2,3,4,5,6,10,19-octanorandrost-8(14)-en-7-oic acid CoA ester; COCHEA-CoA–9-oxo-1,2,3,4,5,10,19-octanor-13,17-secoandrost-8(14)-ene-7,17-dioic acid CoA ester [9,53].

During the degradation of 17β-estradiol by the mixture of strains the new intermediates pent-4-enal; 3-(1,5-dihydroxy-7α-methyl-octahydro-inden-4-yl)-propionic acid and 13-ethyl-3,4,16-trihydroxy-6,7,8,9,11,12,13,14,15,16-decahydro-17H-cyclopenta[α] phenanthne-17-one appeared. Cooperation of the strains allowed 17β-estradiol to break down into non-aromatic products. The mechanisms of estrogen degradation by bacteria are shown in Figure 2 and Figure 3.

Wang et al. [51] described the estradiol degradation strategy that is based on the conversion of estrogens to androgens and the subsequent degradation by the anaerobic 2,3-seco pathway. *Denitratisoma* sp. strain DHT3 converts estrogens into androgens through the cobalamin-dependent methylation of estrogen, including the formation of a nucleophilic carboanion at C-10 on the phenolic A-ring. Androgenic intermediates are catabolized to 9,17-dioxo-1,2,3,4,10,19-hexanorandrostan-5-oic acid (HIP) [51,52].

The *Comamonas testosteroni* strain not only shows the ability to degrade estrogen derivatives, but also testosterone by the 9,10-seco pathway. The first step of testosterone degradation by this pathway is the dehydrogenation of the 17β-hydroxyl group to androst-4-en-3,17-dione, which is converted to androsta-1,4-diene-3,17-dione. The degradation of the sterane ring begins with the introduction of a hydroxyl group at position 9 of the aromatic ring, which leads to the formation of an unstable intermediate. As a result of the B ring cleavage and aromatization of the A ring of this compound, a secosteroid (3-hydroxy-9,10-seco-androsta-1,3,5(10)-triene-9,17-dione) is formed. Further degradation steps include hydroxylation of the A ring in position 4 and its cleavage by 3,4-dioxygenase to 4,5-9,10-diseco-3-hydroxy-5,9,17-trioxoandrosta-1(10),2-diene-4-oic acid (4,9-DHSA) [27]. These compounds may undergo decomposition to

**Figure 4:**
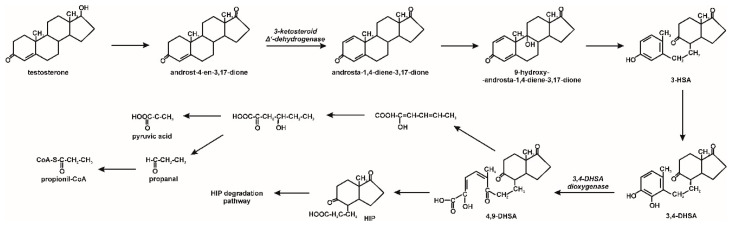
Testosterone biodegradation by bacteria in aerobic conditions; 3-HSA–3-hydroxy-9,19-seco-androsta-1,3,5(10)-triene-9,17-dione; 3,4-DHSA–3,4-dihydroxy-9,10-seco-androsta-1,3,5(10)-triene-9,17-dione; 4,9-DHSA–3-hydroxy-5,9,17-trioxo-4,5-9,10-diseco-androsta-1(10),2-diene-4-oic acid; HIP degradation pathway; HIP–9,17-dioxo-1,2,3,4,10,19-hexanorandrostan-5-oic acid [9,53,54].

2-Hydroxyhexa-2,4-dienoate, and next to compounds incorporated into the central metabolism. Moreover, 4,9-DHSA may be also transformed to HIP. The degradation of this last one leads to the cleavage of both aromatic rings and, as a consequence, to the forming of propionyl-CoA and succinyl-CoA [9,54]. The degradation pathway of testosterone by the *Comamonas testosteroni* strain is shown in Figure 4.

**Figure 5:**
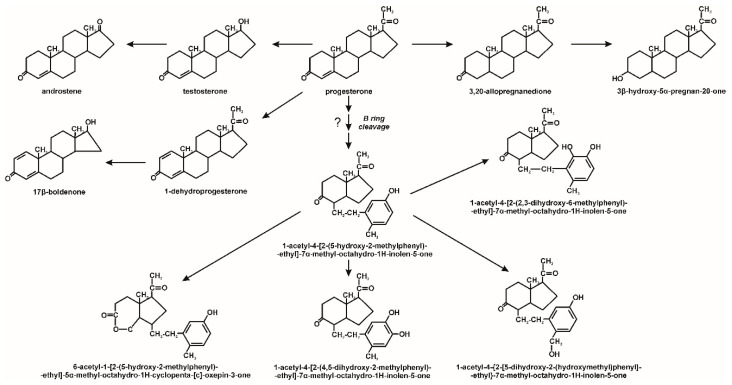
Proposed progesterone biotransformation by algae [55].

A similar pathway has been characterized for progesterone degradation. This compound may be transformed to testosterone after a side-chain breakdown. Moreover, progesterone may undergo hydroxylation, hydrogenation and dehydrogenation to 3β-hydroxy-5α-pregnan-20-one, 3,20-allo-preganedione and 1,4-pregnadiene-3,20-dione, respectively. These compounds were earlier observed during progesterone transformation by microalgae [55]. Ojoghoro et al. [55] also observed the cleavage of the progesterone B ring between C9-C10 to 1-acetyl-4-[2-(5-hydroxy-2-methyl-phenyl)ethyl]-7α-methyl-octahydro-1*H*-inden-5-one (Figure 5).

**Figure 6:**
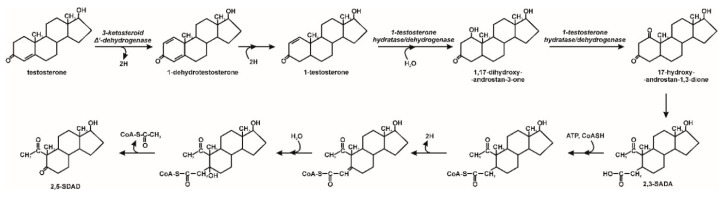
Testosterone biodegradation by bacteria in anoxic conditions; 2,3-SAOA–17-hydroxy-1-oxo-2,3-seco-androstan-3-oic acid; 2,5-SDAD–17-hydroxy-2,5-seco-3,4-dinorandrost-1,5-dione [57].

Testosterone can also be degraded under anaerobic/anoxic conditions by denitrifiers, including *Steroidobacter denitrificans, Sterolibacterium denitrificans* and *Thauera terpenica*. Denitrification of the strain *Steroidobacter denitrificans* oxidized testosterone to 1-dehydrotestosterone and probably also to androst-4-en-3,17-dione under anoxic condition. The NAD^+^-dependent enzyme, responsible for testosterone dehydrogenation is probably similar to 17β-hydroxysteroid dehydrogenases. The compounds obtained are then transformed to androsta-1,4-diene-3,17-dione. This pathway is similar to the testosterone degradation 9,10-seco pathway in aerobic conditions observed in *Comamonas testosterone*, however, due to the lack of oxygen there is no cleavage of the ring with the participation of dioxygenases [56]. However, androgens may also be degraded through the oxygenase-independent 2,3-seco pathway. The key intermediate in this pathway is 1-testosterone, which is dehydrogenated to 1,17-dihydroxy-androstan-3-one and in the next step to 17-hydroxy-androstan-1,3-dione. This compound is probably cleaved to 17-hydroxy-1-oxo-2,3-seco-androstan-3-oic acid (2,3-SAOA). The carboxylic group of 2,3-SAOA at position C-3 is activated by CoA. The C2 side chain at position C-5 of the activated compound is removed via retro-aldol reaction. The aliphatic chain of this compound is degraded by reactions analogous to β-oxidation (Figure 6) [28,51,53,57].

Microalgae remove hormones through three mechanisms—bioadsorption, bioaccumulation and biodegradation/biotransformation. The ability of microalgae to accumulate pollution is related to their cell volume and surface area. In addition, the efficiency of endocrine substance adsorption on cell surfaces depends on the type of hormones. It was shown that more degradation-resistant diethylstilbestrol is easily adsorbed compared to 17β-estradiol. Despite the ability to adsorb and accumulate in the wall of the microalgae, it appears that the main process for hormone removal is biotransformation/biodegradation [11,26]. Enzymes such as peroxidase, glutathione S-transferase and cytochrome P450 are mainly involved in the biotransformation processes of these compounds [11]. Shi et al. [40] observed that 17β-estradiol was converted to estrone under aerobic conditions, whereas under anaerobic conditions, estrone may undergo interconversion to 17β-estradiol.

**Figure 7:**
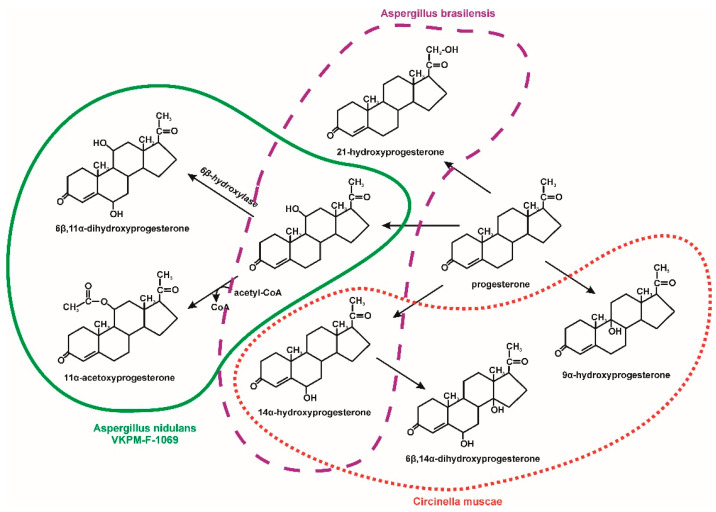
Progesterone biotransformation by fungi [42,45,46].

**Figure 8:**
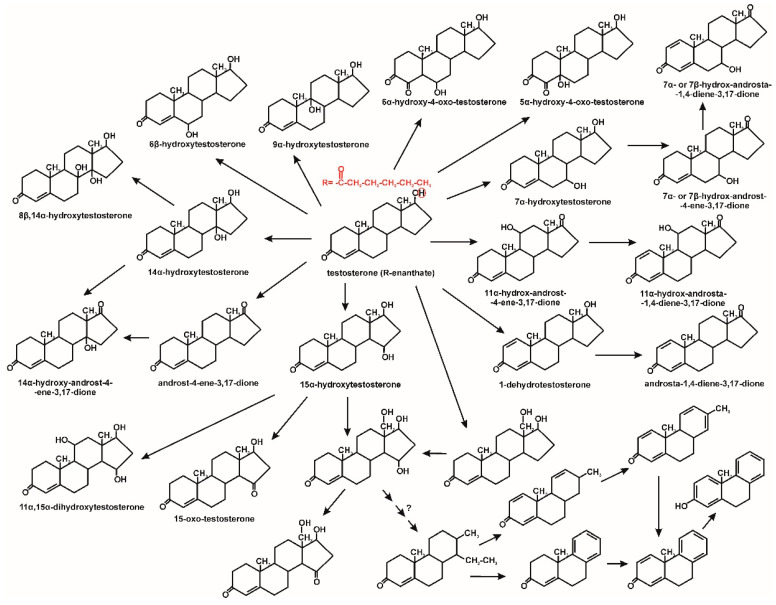
Testosterone and its derivatives biotransformation by fungi [43,44,46].

**Figure 9:**
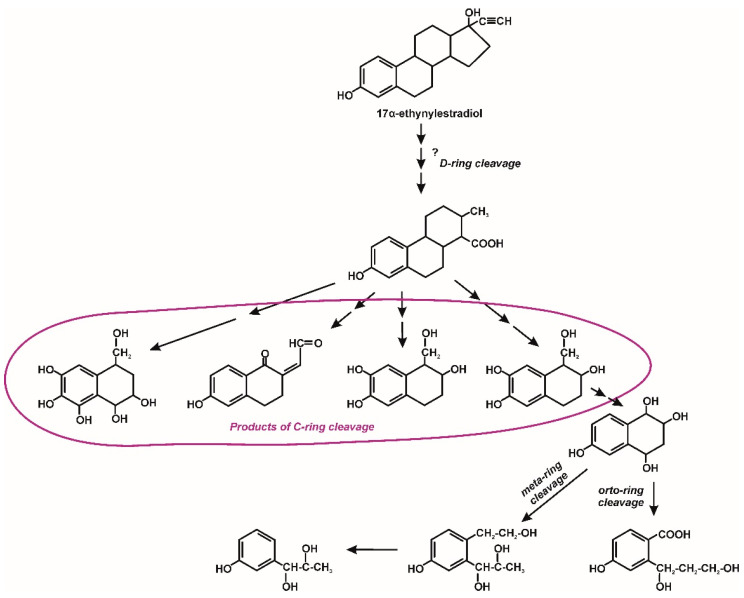
Proposed biotransformation mechanism of 17α-ethynylestradiol by *Lentinula edodes* [44].

Fungal degradation usually occurs using extracellular enzymes. They can interact with mycelium-bound enzymes. Moreover, fungi can secrete low molecular weight redox mediators responsible for increasing the degradation potential. The participation of intracellular enzymes in the degradation of microcontaminants was also observed [30]. An example would be the degradation of 17β-estradiol by *Trichoderma citrinoviride* AJAC3. This fungus secretes ligninolytic enzymes such as manganese peroxidase and lignin peroxidase, which play a significant role in this hormone degradation. In the first stage of degradation, there is a decrease in estrogenic activity due to the conversion of estradiol to estrone. The nature of this reaction is probably radical [22]. During the degradation of EDCs by *Trametes versicolor*, increased laccase activity was observed. The radical mechanism of laccase activity leads to the formation of phenoxy radicals from phenolic EDCs, which undergo nonenzymatic oxidation reactions. Probably, due to the hydrophobic nature of EDCs, intracellular enzymes also participate in their degradation, including the cytochrome P-450 system. The resulting hydroxylated EDCs derivatives can then be metabolized via β-oxidation [30,58]. Hydroxylated derivatives of progesterone were also observed by Hosseinabadi et al. [42] and Zoghi et al. [46] during its biotransformation by *Aspergillus brasiliensis* and *Circinella muscae*, respectively. It was demonstrated that *Aspergillus nidulans* VKPM F-1069 hydroxylates progesterone at the position C11α to 11α-hydroxyprogesterone. Next, this compound undergoes 6β-hydroxylation or acetylation to 6β,11α-dihydroxyprogesterone and 11α-acetoxyprogesterone, respectively (Figure 7) [45]. In turn, *Ascomycota* and *Zygomycota* filamentous fungi are able to effective hydroxylation at position 7α-, 7β-, 11α- and 14α of 3-oxo-4-ene androstane steroids (Figure 8) [43]. Muszyńska et al. [44] identified intermediates produced during testosterone and 17α-ethynylestradiol decomposition by *Lentinula edodes*. On this basis, we have proposed a degradation pathway for 17α-ethynylestradiol, shown in Figure 9.

Little is known about the degradation pathways of mycotoxins and bactericidal compounds with hormonal activity. One of the better described degradation pathways is the decomposition of zearalenone, a compound that is a serious threat because of its presence in contaminated food. This mycotoxin compound is broken down by cleaving the lactone ring to 1-(3,5-dihydroxyphenyl)-10′-hydroxy-1-undecen-6′-one or (5S)-5-({2,4-dihydroxy-6- [(1E)-5-hydroxypent-1-en-1-yl]benzoyl}oxy) hexanoic acid. These intermediates do not have estrogenic activity [31]. Takeo et al. [59] described the 411-nonylphenol degradation pathway by *Pseudomonas putida* KT2440. A side chain of 411-nonylphenol is released from the aromatic ring as a dead-end product–3-methyl-3-octanol as a result of ipso-hydroxylation catalysed by monooxygenase. The second intermediate is hydroquinone, which is assimilated by bacterial cells [59].

It appears that the ability of microorganisms to biotransform EDCs is a universal feature. However, the information presented above shows that only a few microorganisms are capable of completely degrading endocrine compounds. Therefore, it is highly justified to exploit the potential of such strains in purification systems. However, it requires protection of the strains against the competition of indigenous microflora and changing environmental conditions.

## 4. Immobilized Systems in Microbiological Degradation of EDCs

Physico-chemical methods based on materials with a large specific surface and high porosity are used to remove EDC from water and wastewater. The most commonly used materials are zeolite, carbon nanotubes, graphene, mesoporous silica, activated carbon, fullerene or metal-organic frameworks as well as waste materials such as lignocellulose-based hydrochars. The effectiveness of hormone adsorption on these materials depends on many factors, including the nature of the environment, pore filling, and the potential for generated interactions, including electrostatic, hydrophobic, hydrogen and π-π interactions [60,61]. Despite promising results and economic viability, conventional adsorption technology does not solve the problem of EDCS degradation, but only allows for their removal from a given environment. However, the adsorbents require regeneration during which EDCs are removed practically unchanged. Hence, it is justified to search for safe methods allowing for complete degradation of EDCs. The promising results were obtained with usage microorganisms. However the main problem is the sensitivity of functional strains to environmental factors. The limitation for bacteria with increased degradation potential is the competition between these strains and indigenous microflora, and the low tolerance to changing environmental conditions. Protection of microorganisms against negative environmental factors, such as radiation, antibacterial substances, hazardous materials and high osmotic pressure, as well as against competition with an indigenous microbiome and attenuation of phagocytosis is possible due to their immobilization in the matrix of the carrier [4,6]. Currently, the dynamic development of immobilization techniques is observed and new types of carriers, both natural and synthetic, are being designed and introduced [62]. Classical carriers include calcium alginate that has been used for a long time in immobilization [4,41,63,64,65,66], e.g., promising results were obtained by Liu et al. [4], who entrapped the cells of the *Novosphingobium* sp. ARI-1 strain in this matrix. They showed that the immobilized strain had a greater degradation potential than free cells. After immobilization they observed that estrone, 17β-estradiol and estriol were degraded in 80.43%, 94.76% and 100%, respectively. Also, the *Rhodococcus* sp. strain JX-2 had a higher yield of degradation of 17β-estradiol in a harsh environment and removed this compound from wastewater and cow dung after immobilization in calcium alginate [6]. Despite the numerous advantages of this carrier, such as low cost, low toxicity, rapid exchange of nutrients and metabolites, these authors have noticed that the reuse of immobilized microorganisms caused clogging in the pores in the carrier and as the number of cycles increased, the diffusion of oxygen and nutrients was more limited. This feature and the relatively low mechanical strength of this carrier may adversely affect biodegradation processes using strains immobilized in calcium alginate [4]. However, biopolymers could still be successfully used in immobilization, i.e., glutaraldehyde cross-linked chitosan was used to immobilize fungal laccase during bisphenol-A degradation [67]. Chitosan is a natural biopolymer characterized by good biocompatibility, biodegradability, low cost and easy modification, however, the mechanical properties of pure chitosan must be improved for efficient reuse. Nonetheless, the authors used cross-linkages with glutaraldehyde. The additional reaction with the amino polysaccharide of chitosan improved the mechanical resistance of the chitosan beads. Because of its cationic nature, glutaraldehyde stabilizes chitosan solubilization in an aqueous acidic environment as well. The chitosan matrix has achieved great storage stability, 90% of the initial activity, whereas the free enzyme showed only 47.3%, after 28 days at 4 °C. Also, activity after 10 oxidation cycles decrease no more than 30%. Hence, many research centres are looking for new solutions with more stable carriers [67].

Menashe et al. [7] obtained promising results by developing an innovative macro-encapsulation technology, the “small bioreactor platform” (SBP) capsule (patent application No. PCT/IL2010/256). The SBP method is based on the macro-encapsulation of a bacterial culture in a closed environment using a cellulose acetate microfiltration membrane as a protective barrier. The capsule separates the suspended microbial culture inside the capsule from the autochthonous microorganisms in the effluent, while allowing the diffusion of nutrients through the capsule membrane. As a result, the biomass in SBP capsules quickly acclimatizes. In addition, the physical barrier prevents leaching of selective microorganisms from the bioreactor. The *Rhodococcus zopfii* and *Pseudomonas putida* strains encapsulated using this technology degraded 17α-ethynylestradiol with an efficiency of 73.8% and 86.5%, respectively. The authors showed that macro-encapsulation enables the practical application of bacterial cultures in the removal of steroid hormones in wastewater treatment processes. The SBP encapsulation method can be implemented in the sewage treatment plant processes because it overcomes the main obstacles that usually make biological bioaugmentation ineffective: it enables achieving long-term selective biomass implementation in wastewater treatment plants, controls culture location and gives relatively efficient biodegradation that can be synchronized with the hydraulic retention time of the wastewater treatment plant [7]. Gao at al. [41] attempted to used immobilized cells of *Chlorella vulgaris* in nonylphenol removal. The authors achieved over 98% degradation with immobilized beads in the presence of 1 mg/L nonylphenol, but alginate carriers still have many limitations as mentioned above. One of those used in microbial immobilization biopolymers could be cellulose triacetate (CTA). Ma et al. [68] used the entrapment technique with CTA to degrade estrone and 17β-estradiol with two bacteria strains—*Sphingomonas* sp. AHC-F and *Sphingobium* sp. AX-B. Cellulose triacetate is characterized by high hydrate permeation and mechanical strength. CTA presents a great balance of the hydrophilic/hydrophobic character important for effective enzyme adsorption and its biological activity. Additionally, hydrophilic/hydrophobic properties could be modified by acetylation/deacetylation [69]. Both strains were able to degrade 2 mg/L of estrone and 17β-estradiol for free strains in 24 h and for immobilized in 72 h, which is probably caused by the compounds transfer limitation in a porous carrier. Additionally, the authors reported that a continuous-flow degradation on an IBRC reactor of 850 ng/L 17β-estradiol is possible [69].

On the other hand, not only immobilized microorganisms could be used in EDCs degradation. Immobilized enzymes are widely used systems in xenobiotic degradation experiments. Many of the enzymes used are fungal laccases and peroxidases, with a wide range of substrate specificity, high pH and temperature tolerance. Another popular carrier is luffa sponge fibres. The desirability of natural fibres includes complete biodegradability, low cost, renewable nature and relatively high surface area. Lacerada et. al. [70] compared the activity of free and immobilized *Luffa cylindrica* fibres laccases being able to degrade 17α-ethinylestradiol. They showed the possibility of reusing immobilizes laccases systems, despite nonoptimal reaction conditions. It could be a compromise between the degradation level and the possibility of using carriers in many cycles. Laccase, as a wide spectrum oxidative enzyme, was used in many other degradation experiments. The TiO_2_ immobilization technique shows a significant ability to coordinate with amine and carboxyl groups with a good biocompatibility and low price as well. Titanium dioxide also exhibits possibilities for great modification. One of the techniques including TiO_2_ usage is coating by TiO_2_ sol-gel of montmorillonite (O-MMT) electrospun nanofibrous membrane to prepare a functional composite nanofibrous material as a laccase immobilization matrix. Ji et al. [71] reported a significant protective effect of titanium dioxide during immobilization of laccase from *Pleurotus ostreatus*. In that study, the authors used two micro-pollutants, including bisphenol-A known as EDCs compound. Immobilization had a positive influence on the enzyme activity at a pH range of 2.5–7 and stability during 12 h incubation (at pH 3 as a benchmark). Also, the temperature influence on laccase activity was lower in the immobilized system. Both, free and immobilized enzymes work at a temperature range of 25–70 °C, but free laccase reaches a lower activity compared to the immobilized one. It shows that immobilization on advanced carriers may stabilize enzymes and allows the system to reuse them. However, it should be noted that the degradation rate after each cycle was lower. In the 24 h degradation cycle in hybrid membrane reactors (pH 5.5, 25 °C, and 5 L/m^2^ h of flux) Ji et al. (2017) reported a 50% decrease of bisphenol-A decomposition [71].

In a similar experiment, using laccase from *P. ostreatus*, Brugnaria et al. [72] used monoaminoethyl-N-aminoethyl (MANAE-agarose) as a reusable immobilization carrier. Modified agarose carriers like MANAE-agarose are widely used in the physical adsorption of enzymes characterized by good immobilization efficiency with a significant influence on enzyme activity [73]. In comparison to free laccase pH, thermal and storage stability were improved. Both, free and immobilized laccase have optimal activity at pH 5, however, immobilized laccase at pH 8 loses 40% of its initial activity compared to the free enzyme which loses 80%. Significantly, the temperature stability was higher. In comparison to free laccase, relative activity was 2.3- and 7-fold higher at the temperatures of 40 and 55 °C, respectively. Also, storage at 4 °C was much improved by immobilization. After 20 and 40 days, free enzymes lose 50 and 60% of its initial activity, whereas immobilized enzyme loses only 20 and 30% of its initial activity after 40 and 170 days of storage, respectively. It shows the importance of carrier choice to the immobilized system. During another study, Becker et al. [74] showed a high level of EDCs degradation by fungal laccase from *Trametes versicolor* and *Myceliophthora thermophila* immobilized on acrylic beads. After 24 h free *T. versicolor* laccase decomposes 27.8% of 17β-estradiol while the immobilized enzyme degraded 100% of the dose used. Free *M. thermophila* laccase had a better removal rate—63.2%—however, the immobilized system degrades 100% of the 17β-estradiol as well. Experiments with other EDCs, such as bisphenol A (BPA), nonylphenol (NP), benzylbutylphthalate (BBP) or propylparaben (PrPb) show very similar dependence. *T. versicolor* laccase degraded 20.9, 51.5, 1.16 and 8.54% of BPA, NP, BBP and PrPb, respectively, whereas the immobilized enzyme showed removal of 82.0, 81.7, 95.5 and 100%, respectively. For laccase from *M. thermophila* similar results were obtained. Nevertheless, absorption of EDCs on acrylic beads was significant, and in most cases comparable, with the degradation results. The authors correlate a high level of absorption with laccase activity as a positive combination of EDCs removal, however, this point of view could be questioned [74]. Nevertheless, immobilization on nanobeads or nanoparticles became more popular in EDCs degradation as well. Xiao et al. [75] described the immobilization of horseradish peroxidase on Fe_3_O_4_ nanoparticles for the removal of bisphenol-A and 17α-ethinylestradiol. In that case, removal of both the EDCs used was slightly higher in the immobilized system than in the free enzyme solution, however, the authors advised that enzyme immobilized on iron (II, III) oxide could be easily magnetically separated from the solution and reused by contrast to free peroxidase, which is its greatest advantage. After the fifth cycle of using the immobilized enzyme, the removal efficiencies of BPA and 17α-ethinylestradiol were 56% and 48%, respectively. The level of the removal rate is still notable and could be relevant in potential use in the industry, however, it still requires more research. Laccase from *T. versicolor* immobilized on novel polyamide/polyethylenimine (PA/PEI) nanofibers also shows great reusable properties [76] with 100% initial activity after five cycles of 2,2’-azino-bis(3-ethylbenzothiazoline-6-sulfonic acid (ABTS) oxidation. Removal efficiency against triclosan, 17α-ethinylestradiol and bisphenol-A was 73.6, 47.3 and 27.9%, respectively. It should be noted that EDCs removal in wastewater effluent was slightly decreased. Whereas, the storage stability of immobilized laccase after 30 days of storage retained 52% of its initial activity. Despite an almost 50% decrease of immobilized system activity during storage, Maryškova et al. [76] shows great potential to reuse the PA/PEI carrier in continuous cycles of oxidation. It makes PA/PEI polymer potentially useful in EDCs and other pollutant removal. As can be seen, reusability is one of the most important advantages of the immobilization process. Using polystyrene-divinyl benzene (PS-co-DVB) microspheres with poly(glycidyl methacrylate) chains as the enzyme carrier allows the reuse of the enzyme in 10 degradation cycles while retaining its initial activity of about 53% for bisphenol A and 67% for Congo Red dye [77]. Modification of nanoparticle and microparticle materials, such as PS-co-DVB except significant surface areas, gives the possibilities to surface modifications by functionalized groups such as epoxy, hydroxyl, amine, thiol, carboxyl, chloride, bromide or cyclic carbonate. It opens many possibilities to change, modify and optimize immobilization of enzymes and whole cells [77]. Summarize of immobilization matrix/technologies in EDCs biodegradation in Table 3 is presented. The use of natural carriers, such as luffa fibres or biopolymers such as chitosan, is still a widely developed issue. Simultaneously, researchers attempt to modify these carriers by introducing new functional groups or combining techniques such as cross-linkage of chitosan. On the other hand, synthetic carriers, nanoparticles and metals usage increases. Moreover, development of the properties are still the subject of research.

## 5. Conclusions and Perspectives

In the last two decades, there has been a dynamic development of immobilization methods. Many of them have been used in bioremediation research, including the biodegradation of EDCs. Currently, both whole microorganisms are immobilized as well as enzymes involved in the breakdown of the complex structure of EDCs. Despite the many advantages of immobilization, such as prolonged enzyme activity, the protective action of the carrier, the possibility of multiple use, there are still many problems to be solved. A still unresolved problem is the loss of biocatalyst activity in the immobilization process, as well as the hindered diffusion of nutrients and gases into the interior of the carrier. It is extremely difficult to find a carrier that, on the one hand, is characterized by high mechanical strength, and on the other hand, the immobilization process does not damage the biocatalyst and does not interfere with the exchange of nutrients.

More and more attention has been focused on the use of natural carriers characterized by high biocompability, relatively simple synthesis and low price in immobilization. This probably means that natural carriers will still be significant carriers in many processes involving immobilization. However, due to their relatively poor mechanical strength, they are chemically modified to achieve greater stability. The second line of research is the development of the surface of porous materials, as well as the immobilization of biocatalysts on membranes ensuring a large active surface. Such carriers are increasingly used in reactor bio-purification processes. Magnetic or very light carriers, which can be easily separated under reactor conditions, are becoming equally popular. Simultaneously, one of the paths in the search for new carriers is a synthesis of composites and hybrid materials such as poly(acrylic acid), cellulose, polyvinyl alcohol and chitosan or chitosan and alginate, NH_2_-alginate and silica, carbon nanotube–silica composites and many others. A hybrid connection of various materials could reveal products with better properties than each material separately. Recently, carriers based on nanoparticles, including graphene or metal-organic frameworks, have been gaining popularity, which are highly biocompatible and improve the stability of enzymes. It has been shown that the interaction of nanoparticles with enzyme active sites may not only improve the stability of the active site, but may also modify the properties of the active site. Regardless of all current trends, work on the use of immobilized biocatalysts in EDCs bioremediation must be continued because, so far, the experiments have been conducted mainly on a micro scale that does not reflect the real conditions. A new trend in research is the creation of so-called microcosms, the task of which is to imitate natural or anthropogenically changed environments. Maintaining this trend gives hope for the construction of new systems that can be used in the bioremediation of polluted environments. Nonetheless, the in the complex microbial consortia search continues to create new, stable, and efficient carriers for biological treatment such as catalytic processes and biosensors, as well as removing hazardous materials such as EDCs. It is worth mentioning that all the designed carriers should be adapted to a specific enzyme and/or process, because a carrier suitable for one particular catalysis could be highly inefficient to another one.

## Figures and Tables

**Table 1:** Occurrence of EDCs in the environment.

EDCs	Concentration	Occurrence	Sampling Period	References
Estrone	10.4 ng/L	Leca River (Portugal)	Spring to autumn	[4]
17β-Estradiol	5.9 ng/L	Leca River (Portugal)	Spring to autumn	[4]
Estriol	4.4 ng/L	Leca River (Portugal)	Spring to autumn	[4]
Estrone	1.27 ng/L	Wuluo River (Taiwan)	December to May	[12]
17β-Estradiol	313.60 ng/L	Wuluo River (Taiwan)	December to May	[12]
Estriol	210.00 ng/L	Wuluo River (Taiwan)	December to May	[12]
17β-Estradiol	40–117 ng/L	Surface water of Taihu Lake, China	May	[13]
17β-Estradiol	9.78–151 ng/L	Sediments of Taihu Lake, China	May	[13]
17β-Estradiol	0.076–0.233 ng/L	Lake Balaton, Hungary	No data	[14]
17α-Ethynyl-estradiol	0.68 ng/L	River Zala, Hungary	No data	[14]
Estrogen	3.22–20.61 ng/L	Yangtze estuary, China	Four seasons	[15]
Progesterone	20.4–439 ng/L	Themi River, Tanzania	March	[16]
Progesterone	16,689 ng/L	Kalansanan River, Malaysia	No data	[16]
Androstenedione	40 ng/L	Fenholloway River, USA	May and December	[17]
Progesterone	2060 ng/L	Fenholloway River, USA	May and December	[17]
17α-Ethynyl-estradiol	981–4390 ng/L	Atibaia River, Brazil	High rainy periods	[18]
17α-Ethynyl-estradiol	17–501 ng/L	Atibaia River, Brazil	Dry winter period	[18]
17β-Estradiol	464–6806 ng/L	Atibaia River, Brazil	High rainy periods	[18]
17β-Estradiol	106–2273 ng/L	Atibaia River, Brazil	Dry winter period	[18]
Estrone	16 ng/L	Atibaia River, Brazil	High rainy periods and dry winter period	[18]
Progesterone	20 ng/L	Atibaia River, Brazil	High rainy periods	[18]
Progesterone	20–195 ng/L	Atibaia River, Brazil	Dry winter period	[18]
Levonorgestrel	19 ng/L	Atibaia River, Brazil	High rainy periods	[18]
Levonorgestrel	19–663 ng/L	Atibaia River, Brazil	Dry winter period	[18]
4-Octylphenol	21 ng/L	Atibaia River, Brazil	High rainy periods and dry winter period	[18]
4-Nonylphenol	18 ng/L	Atibaia River, Brazil	High rainy periods and dry winter period	[18]
Testosterone	1.9–2.7 ng/L	Lower Jordan River	May	[19]
Testosterone	1.0–3.8 ng/L	Lower Jordan River	October	[19]
Estrogen	0.6–2.2 ng/L	Lower Jordan River	May	[19]
Estrogen	1.2–3.4 ng/L	Lower Jordan River	October	[19]
Estriol	0.9–1.8 ng/L	Lower Jordan River	May	[19]
Estriol	0.9–2.9 ng/L	Lower Jordan River	October	[19]

**Table 2:** Microorganisms engaged in EDCs biodegradation/biotransformation.

Organism	EDCs	Dose	Efficiency [%]	References
*Virgibacillus halotolerans*	17β-estradiol	5 mg/L	100	[3]
*Bacillus flexus*	17β-estradiol	5 mg/L	100	[3]
*Bacillus licheniformis*	17β-estradiol	5 mg/L	100	[3]
*Novosphingobium* sp. ARI-1	estrone	1.75 µg/L	80.43	[4]
	estriol	1.52 µg/L	100	[4]
	17β-estradiol	0.71 µg/L	94.76	[4]
*Rhodococcus* sp. JX-2	17β-estradiol	30 mg/L	94	[6]
*Rhodococcus zopfii*	17α-ethynylestradiol	2 mg/L	86.5	[7]
*Pseudomonas putida* F1	17α-ethynylestradiol	0.5 mg/L	73.8	[7]
*Acinetobacter* sp. DSSKY-A-001	17β-estradiol	40 mg/L	90	[10]
*Acinetobacter* sp. LHJ1	17β-estradiol	0.5 mg/L	100	[10]
*Acinetobacter* sp. BP8	17β-estradiol	1.8 mg/L	100	[10]
*Acinetobacter* sp. BP10	17β-estradiol	5 mg/L	100	[10]
*Novosphingobium* sp. SLCC	estrone	1 mM	no data	[12]
*Sphingomonas* sp. KC8	estrone	0.05 mg/L	100	[25]
	17β-estradiol	0.05 mg/L	100	[25]
	testosterone	0.05 mg/L	100	[25]
*Thauera* sp. GDN1	testosterone	1 mM	no data	[28]
*Rhodococcus pyridinivorans* K408	zearalenone	5 mg/L	87.21	[31]
*Bacillus* sp.	17β-estradiol	0.2 mg/L	91.70	[33]
*Aeromonas punctata*	17β-estradiol	0.2 mg/L	94.20	[33]
*Klebsiella* sp	17β-estradiol	0.2 mg/L	100	[33]
*Enterobacter* sp. I	17β-estradiol	0.2 mg/L	77.10	[33]
*Enterobacter* sp. II	17β-estradiol	0.2 mg/L	85.40	[33]
*Aeromonas veronii*	17β-estradiol	0.2 mg/L	93.40	[33]
*Acinetobacter* sp. LM1	17β-estradiol	5 mg/L	77.00	[34]
*Pseudomonas* sp. LY1	17β-estradiol	5 mg/L	68.00	[34]
*Sphingomonas* sp. MCCC 1A06484	17β-estradiol	1.8 mg/L	78.70–98.80	[35]
*Comamonas testosterone* ATCC1199	testosterone	0.5 mM	60.00	[36]
	17β-estradiol	0.5 mM	35.00	[36]
	estrone	0.5 mM	45.00	[36]
	estriol	0.5 mM	25.00	[36]
*Comamonas testosteroni*QYY20150409(CT)	17β-estradiol	1 mg/L	76.00	[37]
*Rhodococcus equi* DSSKP-R-001(RS)	17β-estradiol	1 mg/L	86.00	[37]
*Deinococcus actinosclerus* SJTR1	17β-estradiol	10 mg/L	90.00	[38]
*Stenotrophomonas maltophilia* SJTL3	17β-estradiol	10 mg/L	90.00	[39]
*Raphidocelis subcapitata*	17β-estradiol	1.5 mg/L	74.60	[11]
	diethylstilbestrol	1.5 mg/L	54.10	[11]
*Chlorella vulgaris*	testosterone	0.2 mg/L	69.64	[26]
*Chlorella vulgaris*	nonylphenol	1 mg/L	93.00	[41]
*Trichoderma citrinoviride* AJAC3	17β-estradiol	200 mg/L	99.60	[22]
*Trametes versicolo*	dimethyl phthalate	100 µM	60.00	[30]
	methyl paraben	100 µM	100.00	[30]
	butyl paraben	100 µM	100.00	[30]
	nonylphenol	100 µM	85.00	[30]
	bisphenol A	100 µM	100.00	[30]
*Pleurotus ostreatus*	dimethyl phthalate	100 µM	50.00	[30]
	methyl paraben	100 µM	100.00	[30]
	butyl paraben	100 µM	98.00	[30]
	nonylphenol	100 µM	68.00	[30]
	bisphenol A	100 µM	60.00	[30]
*Phanerochaete chrysosporium*	dimethyl phthalate	100 µM	65.00	[30]
	methyl paraben	100 µM	69.00	[30]
	butyl paraben	100 µM	58.00	[30]
	nonylphenol	100 µM	69.00	[30]
	bisphenol A	100 µM	66.00	[30]
*Aspergillus brasiliensis*	progesterone	10 mg/L	no data	[42]
*Absidia coerulea* VKM F-833	androst-4-ene-3,17-dione	1 g/L	29.00	[43]
*Beauveria bassiana* VKM F-2533	androst-4-ene-3,17-dione	1 g/L	65.00	[43]
	androsta-1,4-diene-3,17-dion	1 g/L	30.00	[43]
*Drechslera* sp. Ph F-34	androst-4-ene-3,17-dione	1 g/L	4.00	[43]
	androsta-1,4-diene-3,17-dion	1 g/L	8.00	[43]
	testosterone	1 g/L	100.00	[43]
*Gibberella zeae* VKMF-2600	androsta-1,4-diene-3,17-dion	1 g/L	7.00	[43]
*Cunninghamella echinulata* VKM F-439	androst-4-ene-3,17-dione	1 g/L	6.00	
*Lentinula edodes*	testosterone	200 mg/L	no data	[44]
	17α-ethynylestradiol	0.8 mg/L	no data	[44]
*Aspergillus nidulans* VKPM F-1069	progesterone	1 g/L	no data	[45]
*Circinella muscae*	progesterone	7 g/L	no data	[46]
	testosterone enanthate	7 g/L	no data	[46]

**Table 3:** Immobilization matrix/technologies in EDCs biodegradation.

Immobilization Matrix/Technology	Pros and Cons of Matrix/Technology	Microorganism/Enzyme	EDCs	References
Alginate/CaCl_2_	+ increased degradation efficiency and/or enzyme activity + better drug tolerance + improved thermal stability of enzymes + low cost + non-toxic carrier + simple technology + favourable mass transfer characteristics +/− high mechanical stability − poor recycling and reuse properties − risk of pore occupation and diffusion limitation during system reuse − unstable in the presence of phosphate − possibility of compound sorption on/in carrier	*Novosphingobium* sp. ARI-1 *Rhodococcus* sp. JX-2 *Chlorella vulgaris* *Cyberlindnera fabianii* (laccase)	estrone estriol 17β-estradiol 17β-estradiol nonylphenol bisphenol A	[4] [6] [41] [66]
Alginate/CaCl_2_ + α-cyclodextrin (α-CD)	+ better degradation efficiency compared to non-α-CD alginate beads + better drug tolerance + low cost + simple technology + favourable mass transfer characteristics + high mechanical stability +/− strong affinity for nonylphenol − use of toxic compounds during process (e.g., cyanogen bromide) − poor recycling and reuse properties − degradation slower than freely-suspended cells − risk of pore occupation and diffusion limitation during system reuse	*Sphingomonas cloacae*	nonylphenol	[32]
	possibility of compound sorption on/in carrier			
Encapsulation in small bioreactor platform (SBP)	+ better biodegradation capabilities + high physically separates + high permeability cellulose acetate microfiltration membrane + non-toxic carrier + relatively simple technology + low carrier absorption level	*Rhodococcus zopfii* *Pseudomonas putida* F1	17α-ethynylestradiol 17α-ethynylestradiol	[7]
Loofah (*Luffa* spp.) sponge	+ natural carrier + low cost + simple technology + biodegradable carrier + high porosity − poor reuse properties − low mechanical durability	*Pleurotus ostreatus* (laccase)	17α-ethinylestradiol	[70]
Chitosan/glutaraldehyde	+ good stability + possibility to reuse system + good mechanical durability + increased enzyme stability during storage + increased enzyme thermal stability + low cost carrier + simple technology + non-toxic carrier	*Trametes versicolor* (laccase)	bisphenol-A	[67]
Cellulose triacetate	+ high hydrate permeation + high mechanical durability + high porosity + biodegradability potential − slight degradation slower than freely-suspended cells	*Sphingomonas* sp. AHC-F *Sphingobium* sp. AX-B	17β-estradiol estrone 17β-estradiol estrone	[68]
Titania nanoparticles	+ low price + good chemical stability + good biocompatibility + chemical coordination ability + high reuse potential + great carrier modification potential + increased enzyme stability in various pH + increased enzyme stability at various temperatures	*Pleurotus ostreatus* (laccase)	bisphenol A carbamazepine	[71]
MANAE-agarose	+ great reuse potential + increased degradation efficiency + increased enzyme stability during storage + increased enzyme thermal stability + simple technology + low cost + effective against surface carboxyl group particles + increased degradation efficiency	*Pleurotus ostreatus* (laccase)	bisphenol A	[72]
Acrylic beads (IB-EC-1)	+ good enzyme absorption on carrier + possibility to use low enzyme amount	*Trametes versicolor* (laccase) *Myceliophthora thermophila* (laccase)	steroids and EDCs mixture ^1^	[74]
Ceramic membrane	+ good enzyme absorption on carrier − very low degradation efficiency	*Trametes versicolor* (laccase)	steroids and EDCs mixture ^1^	[74]
Fe_3_O_4_ nanoparticles	+ possibility to simple carrier recycling in magnetic field + high reuse possibilities + increased degradation efficiency + low toxicity + good biocompatibility + increased enzyme stability during storage + increased enzyme thermal stability	horseradish peroxidase	bisphenol-A 17α-ethinylestradiol	[75]
Polyamide/Polyethylenimine Nanofibers	+ simple technology + low toxicity + high stability in the environment + safe and easy to handle + presence of plenty of surface amino groups + increased enzyme stability during storage	*Trametes versicolor* (laccase)	bisphenol A 17α-ethinylestradiol triclosan	[76]
Cyclic carbonate group on polymeric microspheres [PS-co-DVB-g-P(CCMA)]	+ high amount of enzyme binding sites on carrier (cyclic epoxy and cyclic carbonate groups) + wider pH and temperature range of enzyme activity + increased enzyme thermal stability + increased enzyme stability during storage − advanced carrier synthesis − use of toxic compounds during process	*Trametes versicolor* (laccase)	bisphenol-A	[77]

^1^ Steroids: estrone, 17β-estradiol, 17α-ethinylestradiol, estriol; EDCs: bisphenol A, nonylphenol, propylparaben, benzyl-n-butyl phthalate.
